# Automatic Image Generation Pipeline for Instance Segmentation of Deformable Linear Objects

**DOI:** 10.3390/s23063013

**Published:** 2023-03-10

**Authors:** Jonas Dirr, Daniel Gebauer, Jiajun Yao, Rüdiger Daub

**Affiliations:** Institute for Machine Tools and Industrial Management, Technical University of Munich, Boltzmannstraße 15, 85748 Garching, Germany

**Keywords:** data-centric AI, machine vision, synthetic images, deformable one-dimensional objects, domain randomization, cable, wire

## Abstract

Robust detection of deformable linear objects (DLOs) is a crucial challenge for the automation of handling and assembly of cables and hoses. The lack of training data is a limiting factor for deep-learning-based detection of DLOs. In this context, we propose an automatic image generation pipeline for instance segmentation of DLOs. In this pipeline, a user can set boundary conditions to generate training data for industrial applications automatically. A comparison of different replication types of DLOs shows that modeling DLOs as rigid bodies with versatile deformations is most effective. Further, reference scenarios for the arrangement of DLOs are defined to generate scenes in a simulation automatically. This allows the pipelines to be quickly transferred to new applications. The validation of models trained with synthetic images and tested on real-world images shows the feasibility of the proposed data generation approach for segmentation of DLOs. Finally, we show that the pipeline yields results comparable to the state of the art but has advantages in reduced manual effort and transferability to new use cases.

## 1. Introduction

Deep learning requires large amounts of labeled training data, hence the lack of data is a current and significant challenge [1]. Large image datasets are available, especially for autonomous driving applications and household scenarios, but manual real-world image acquisition and labeling of new datasets involve considerable time and effort [2]. In particular, annotating segmentation masks is laborious and requires much more time than labeling bounding boxes. Other challenges, when using real-world training data, can be inconsistent label quality and underrepresentation of rare events in the dataset [2]. Synthetic image data generation and an integrated computation of associated labels can compensate for these challenges. Thus, the manual effort can be reduced significantly compared to manual labeling and is no longer directly proportional to the number of training images and annotations.

Synthetic training data for deep learning can be particularly suitable in the industrial context for several reasons [3]. On the one hand, few industrial datasets are publicly available because data is rarely shared due to confidentiality. On the other hand, machine vision tasks, which often occur in a restricted environment with specific boundary conditions, are relevant so generalized problem-solving is not always targeted. In the industrial context, geometric 3D models of parts are usually available to generate synthetic image data. However, due to the varying shape of DLOs, no geometric model is typically available for objects such as cables, single wires, and hoses. Nevertheless, the localization of DLOs is necessary to perform position-flexible and reactive handling and assembly tasks.

In this context, we introduce an automatic data-generation pipeline for DLOs, that builds up on the simulation and rendering method for rigid objects. This pipeline outputs synthetic images of DLOs and corresponding annotations for deep learning in industrial applications. The advantage of the pipeline is that the synthetic image generation of DLOs is flexible in terms of DLO type, shape, arrangement, and use case. In addition, it can automatically generate precise annotations for machine vision tasks such as semantic and instance segmentation. The application of the pipeline does not require geometric 3D models of the DLOs as input. Instead, it allows an operator to describe the boundary conditions of the considered use case through user input. Furthermore, the pipeline limits the manual effort such that it does not depend on the number of generated image data. This enables the cost-efficient generation of large image datasets for DLO-specific applications in the industrial context.

## 2. State of the Art

Regarding the shortage of annotated images for deep learning, two strategies are distinguished. On the one hand, there are approaches to reduce the required data volume and use existing smaller datasets efficiently. On the other hand, approaches exist to create new synthetic training data. In the following, we present an overview of both strategies and then elaborate on data generation methods. Subsequently, works that apply segmentation to DLOs and show the demand for DLO-specific training data are briefly introduced. Finally, we conclude on the state of the art and formulate our contribution within this work.

### 2.1. Efficient Dataset Usage

For the first strategy, according to [4] multiple approaches are used: firstly, deep learning networks can be pre-trained on large freely available datasets such as COCO [5], ImageNet [6], and Pascal VOC [7]. The goal is to initially learn low-level features of a wide range of objects before training on the actual dataset [4]. Secondly, pre-trained networks are fine-tuned for use in a context similar to the original one. Therefore, only the last layers of a network are trained with new data [8]. Thirdly, data augmentation is used to alter images, for example by geometric transforms of objects and pixel operations [9]. The augmentation is applied to increase the size and variability of an existing dataset efficiently.

The interested reader might refer to [10] for a survey on data augmentation. Occasionally, data augmentation is categorized as a data generation method [9]. However, augmentation can only alter existing images and cannot create new images with novel content. Therefore, we do not categorize image augmentation as a method for synthetic data generation but as a strategy to alter existing data [8].

### 2.2. Generation of Synthetic Training Data

Due to the large number of approaches and methods for generating synthetic image data, we focus on non-learning methods to generate datasets for high-level computer vision tasks, such as object recognition and segmentation, that automatically compute corresponding labels. Within this constraint, we distinguish the methods: cut–paste method, simulation and rendering, and hybrid methods. The interested reader might refer to [9], who categorize and assess methods within our scope. Nikolenko [8] provides a comprehensive review of further approaches.

In deep learning, the so-called domain gap can occur when training and test data have different distributions or properties. This gap is challenging in using synthetic training data for training and real-world data for testing. The following sections also address how each data generation method can address the domain gap.

#### 2.2.1. Cut–Paste Method

In the cut–paste method, synthetic images are generated by cutting out objects from real-world 2D images and combining them with new background images. During the cut step, objects are cropped manually [11], semi-automatically [12], or by applying trained neural networks [13] from the source images. These cut objects are fused into new background images during the paste step. Consequently, the corresponding labels are derived automatically. Cropping objects from images and pasting them into new backgrounds can create optical artifacts. Therefore, various augmentation techniques are used to prevent them [13]. A significant advantage of this method is that real-world images out of the target domain are used as source images, which can result in a narrower domain gap [14]. Since the source images are typically generated manually and, in some cases, cropped manually, this method can require extensive manual effort. In addition, the diversity of the generated dataset is limited by the source images, e.g., with regard to lighting and perspective representation [14].

Zanella et al. [12] present an automatically generated dataset for semantic segmentation of DLOs. The DLOs are imaged in front of a background of known color and cropped using chroma-key technology. The cropped DLO images are inserted in front of various background images, and semantic masks are derived. Models trained on this synthetic data demonstrate the effectiveness of their approach for semantic segmentation during validation on real-world data of varying complexity.

#### 2.2.2. Simulation and Rendering Method

For simulation and rendering methods, geometric 3D models of the objects are required. The models are loaded into a simulation to compose 3D scenes optionally with distractor objects. The scene composition can be based on random positioning [15], global parameters [16], or result from physics simulation [17]. These different types of positioning can result in unrealistic arrangements [14] or patch-level realism [1] as well as physically plausible global scene layouts [16,17].

In the second step of the method, 2D images are rendered from viewpoints in the simulation, and corresponding labels are derived automatically. In rendering, parameters are required to define lighting, object texture, and camera settings. The function sequence of the simulation and rendering method can be implemented with programs such as Unreal Engine, Unity, and Blender. Frameworks such as BlenderProc [18] and Kubric [19] exist to support the generation of realistically looking images in these programs.

Two fundamental approaches exist to narrow the domain gap with the simulation and rendering method: domain randomization and realism. In domain randomization, various and even unrealistic expressions of scenes are generated to generalize models to the real world by showing enough variability in the training data [20]. In their early works, Tremblay et al. [21] apply domain randomization to the objects and distractors and show that networks trained on synthetic images can outperform real datasets. Other approaches randomize only selected factors during rendering such as the lighting but apply realistic textures to the objects [15].

Highly realistic and physically accurate images are generated in [22] by using procedural modeling and physically based simulation of light transport. The procedural modeling follows the geometric shape and material properties rules to generate scenes within the simulation. These rules can be defined for each object class individually and allow precise control over the variation form.

Eversberg and Lambrecht [1] compare object detection results for synthetic training data with realism versus domain randomization in the industrial context. Therefore, they contrast both strategies, especially for the aspects of the rendering process, such as lighting, background, object texture, usage of distractors, and label computation. They find that realistic settings with a high variability perform best for object-related properties (e.g., lighting and texture). In contrast, for non-object-related aspects (e.g., distractor objects and background), the concept of domain randomization achieves better scores. Based on these results, domain-specific knowledge can be used in an industrial context to generate artificial training images.

Grard [23] shows one of the few approaches explicitly considering deformable objects. It considers packaged food products in bulk scenarios with high object density. The scene generation uses mesh templates superimposed with textures to simulate various food products. This approach induces random deformation to the geometric template to extend the range of simulated intra-class variation caused by object deformation.

#### 2.2.3. Hybrid Method

In addition to the previous approaches, a hybrid method exists that combines both previous approaches in a two-step procedure. First, a simulation is used to render 2D images from different viewpoints of a 3D model of an object. These images are merged into background images through pixel-based operations, analogous to the second step of the cut–paste method. Thereby, no real-world source images of the object are required. In contrast to the cut–paste method, the method is not only suitable for object recognition [14] and segmentation [24], but also for pose estimation [25], since the pose label can be derived during rendering. Blurring the object and its surrounding, as well as different blending modes, can improve the detection performance analogous to the cut–paste method.

### 2.3. Segmentation of DLOs

The detection or pose estimation of the DLOs’ rigid components (e.g., connectors) [26] is insufficient in many industrial applications because the detection of the entire cable is required to prevent entanglement. For the segmentation of entire DLOs in 2D images, specific models and algorithms for segmentation are proposed, and general models are applied. In particular, learning methods are used to detect DLOs in complex scenes.

In [27] the instance segmentation algorithm Ariadne for DLOs is proposed. The two-stage process requires training data only in the first step for the pre-detection of cable ends. The actual segmentation of the DLOs takes place based on superpixelation and comparing their visual similarity. This approach is developed further to Ariadne+ in [28], where a trained model is applied for the semantic segmentation of DLOs in the first step. Subsequently, graph operations are applied to distinguish individual objects and resolve the overlapping DLOs. The model for the semantic segmentation is trained based on the synthetic dataset generated in [12]. Song et al. [29] propose a method for the topological segmentation of DLOs in cluttered scenes. Two models, one detecting intersections and one segmenting the DLO shape, are trained with manually annotated images and applied to real-world data.

### 2.4. Summary and Contribution

Various applications in the handling technology [30] and assembly may require the localization or segmentation of DLOs. Currently, general-purpose and DLO-specific models are used for such segmentation tasks. First approaches for instance segmentation show promising results [28]. In contrast to the segmentation, the generation of synthetic training data is considered only to a limited extent. The approach of Zanella et al. [12] generates semantic masks but not instance masks and is dependent on acquiring real-world source images. The imaging is accompanied by substantial work in transferability to new use cases regarding DLO types and arrangement. Caporali et al. [28] state the lack of available datasets as a significant problem for applying instance segmentation to DLOs. Grard [23] presents one of the few approaches explicitly considering deformable objects in the simulation method. However, the packaged food products considered are predominantly subject to volume change as opposed to bending deformation typical for DLOs. In addition, no comparison of modeling methods is made.

In order to compensate for the lack of training data for DLOs we provide the following contributions: (1) We introduce an automatic data-generation pipeline for DLOs and demonstrate its validity for multiple object types and use cases. (2) Further, we classify three scenarios for the arrangement of DLOs and provide annotated real-world data for them. (3) On this basis, we compare the segmentation performance of different replication types of DLOs using real-world test images. (4) Lastly, we benchmark our approach with a state-of-the-art approach for synthetic data generation for DLOs.

## 3. Methodology

In this paper, we follow a four-step procedure for the data generation and model training: (1) synthetic training data generation, (2) model training, (3) model test, and (4) evaluation. This procedure is performed multiple times to generate different synthetic datasets with the proposed automatic data-generation pipeline and use them for model training and testing under constant settings. The goal of this procedure is to compare different data generation approaches and to identify suitable types for replicating DLOs for production engineering applications.

In this section, we focus on the methodology for the training data generation. Therefore, we introduce an automatic data-generation pipeline and describe its modules. The data-generation pipeline’s implementation and the remaining procedure are described in Section 4 for specific application scenarios.

### 3.1. Automatic Data-Generation Pipeline

**Figure 1:**
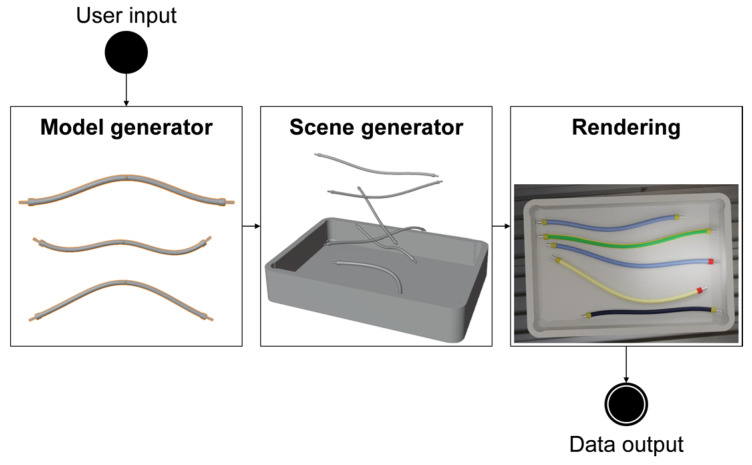
Illustration of the three-step automatic data-generation pipeline for DLOs.

Our data-generation pipeline aims to automatically generate image data of DLOs and associated labels for deep learning. This pipeline should generate images of specific industrial application scenarios with a predefined variability. Thereby, manual efforts for the generation of training data should be minimized. By applying the pipeline, it should no longer be necessary to set up real-world scenes, photograph them, and manually label image data. Building upon the state of the art of the simulation and rendering method, we present an automatic data-generation pipeline (Figure 1) to meet the previous requirements. This enables the generation of synthetic training images for DLOs analogous to the existing approaches for rigid objects.

This data-generation pipeline requires user input that specifies the distribution of the dataset and boundary conditions, for example, the geometric form and visual appearance of the DLOs. Following the *user input*, the first module *model generator* can be executed. It generates geometric models of DLOs with four different replication types, which lead to different levels of realism. In the second module, *scene generator*, previously generated DLO models are selected and placed in a simulation to obtain scenes within the considered distribution. In the third and last module, 2D images are *rendered* from the simulation’s 3D scenes, and the corresponding labels are computed. The output of the method are synthetic 2D images and corresponding label images.

The three modules of the pipeline are repeated automatically to generate variable scenes and multiple individual synthetic images of DLOs. The novelty of this pipeline is characterized by its first module and additional features in the second module. This enables to apply the simulation and rendering method for DLOs. In the following, our focus is primarily on the model generator and the comparison of different replication types (Section 3.2) as well as the scene generator (Section 3.3). An in-depth examination of object textures and rendering parameters is beyond the scope out this publication.

### 3.2. Model Generator

The task of the model generator is to create, based on the user input, models of DLOs that can be utilized for the scene generation. An ideal model would mimic the deformation behavior of a real DLO to achieve the best segmentation results. In addition, the model could be generated based on information readily available such as geometric measures and information from data sheets, and therefore requires little or no expert knowledge in its application. Further, an ideal model would require minimal computational effort during the simulation. In summary, a trade-off between realism, user input, and computational cost is required due to the number of required training images. Therefore, we introduce and compare four ways to replicate DLOs for the synthetic data generation with different grades of realism and complexity:

(a) **Minimal replication:** For the minimal replication, only one rigid body model is generated and used in all scenes. The minimal replication does not show any deformation such that only one straight cable is generated.
(b) **Reduced replication:** The reduced replication is based on the generation of only one rigid body model, too, but with a specific deformation. This geometric model is used with the same shape in all generated scenes. This meets state-of-the-art approaches, where the same model of rigid objects is used repeatedly in the scene generation.
(c) **Visual replication:** For the visual replication, multiple rigid body models are generated. To mimic the typical appearance of deformed DLOs, each DLO model is created with an individual shape. In the simulation, the rigid body models maintain their shape and do not deform depending on the environment. This allows to imitate the visual appearance of deformed DLOs in a quasi-static state without simulation of the actual physical state.
(d) **Physical replication:** In the physical replication, the DLOs are modeled as soft bodies. The objective is to simulate the deformation of the DLOs in a physically possible way. For this purpose, the soft body models are generated with an individual initial deformation. In the subsequent simulation, the DLO models undergo further deformation due to contact with the environment and gravity.

**Figure 2:**
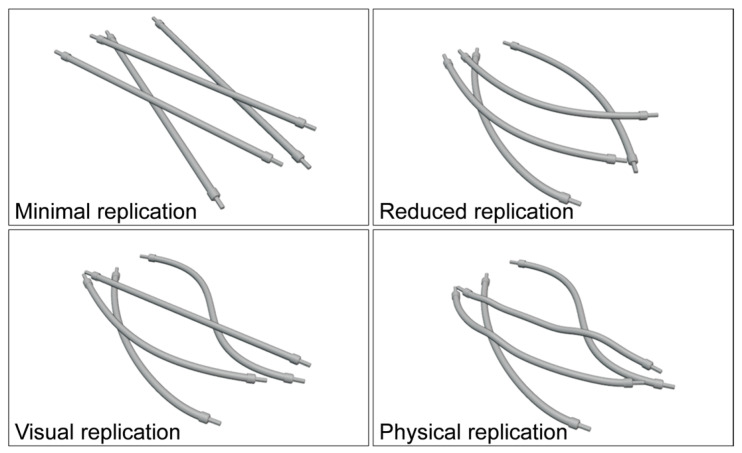
Applied replication types in overlapping scenarios.

Figure 2 shows an overview of the four replication types within scenes where control cabinet cables overlap. These four replication types require different sets of parameters to generate DLO models. The basic shape of a DLO is described by the geometric parameters such as diameter and length. The deformation parameters define the deviation of a DLO from a straight shape. For this, the deformation of a DLO is defined by a relative shift of the control points of the underlying centerline spline. Lastly, the soft body parameters summarize several material and environmental properties that affect the physical deformation of a DLO.

The application of the parameter sets for each replication type is described in Table 1. There it is distinguished whether discrete values or ranges of values can be set for the respective parameters. The parameters required for the respective replication types are provided through the user input in the data-generation pipeline. If a user specifies a parameter space, values are randomly selected within this range during the repeated application of the model generator. Exemplary for the visual replication, this results in geometric models with individual deformations.

DLOs in the industrial context are often not purely deformable objects. In particular, rigid components such as connectors are often present at the ends of the DLO. Regardless of the replication type, a geometric object model (e.g., a CAD model) is used for these components and attached to the ends of the DLO model.

In summary, the model generator can generate DLO models in four different ways. The replication types differ regarding realism, variability, and the required input parameters. Through the input parameters, a user can specify the expected DLO types and generate specific models for them.

### 3.3. Scene Generator and Rendering

Within the data-generation pipeline, the scene generator composes semi-random arrangements of DLOs using a simulation. In addition, we introduce a classification of scenarios for the arrangement of DLOs and describe the model generator in detail. DLOs can be arranged in many different ways. Thus, we define three general scenarios to classify the arrangement of DLOs. These scenarios are based on production-related applications, e.g., where DLOs are supplied to an automatic handling or assembly system. In all three scenarios, the DLO shape is arbitrary, and the DLO pose is variable within a predefined region of interest, e.g., a load carrier. However, the scenarios differ in terms of the number of objects and the distance relation between the objects:

**S1**  Individual provision: One single DLO is provided in the region of interest.
**S2**  Semi-structured provision: Multiple DLOs are provided in the region of interest, but they do not overlap.
**S3**  Unstructured provision: In a region of interest, multiple DLOs are provided and may overlap or cross each other.

**Figure 3:**
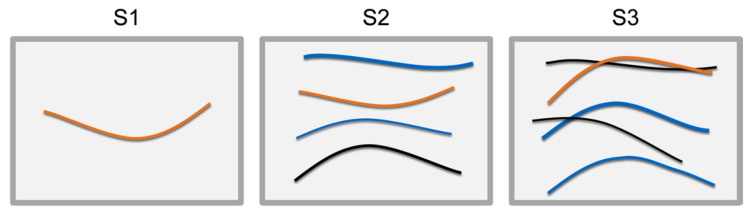
Representation of the three reference scenarios for the DLO arrangement.

Figure 3 shows a representation of the three scenarios. We do not consider DLOs coiled up, DLOs that overlap themselves and knot-like arrangements of DLOs. The scenarios are generated by the scene generator, described in the following, and applied in Section 4 to depict multiple industrial use cases.

Based on the user input, the scene generator generates scenes of the selected scenario within predefined boundary conditions, such as the maximum number of DLOs within one scene. In the first step of the scene generator, industrial periphery objects such as load carriers are placed in the simulation. Subsequently, DLO models are randomly selected from the model generator, loaded into the simulation, and positioned above the region of interest. Consequently, the simulation is run to make the DLOs drop into the scene and create variable setups within the predefined scenario. The initial poses of the DLO models above the scene are decisive for the final arrangement. Additionally, the distribution of DLOs within the scene strongly depends on the simulation settings, such as DLO deformation behavior and friction coefficients. We experimentally determined suitable parameters for the generation of the three scenarios. If applicable to the use case, distractor objects are placed within the scene.

If scenes are created with the visual or physical replication, multiple DLO models with different diameters and lengths can be used. This allows the creation of mixed-type scenarios. In contrast, the same geometric model is used in all scenes for the minimal and reduced replication. Therefore, the scenes cannot represent mixed-variety scenarios via the geometric shape but only via a texture applied downstream.

The rendering in our pipeline builds on the preliminary work described in Section 2 and, in particular, on [1]. Similar to the previous approaches, camera and lighting settings can be selected by the user or randomized. Furthermore, we apply texture to all peripheral objects and the DLOs individually. Thus, a variety of variants is generated through optical features. The rendering results and, thus, the data-generation pipeline output are a dataset of 2D images and the corresponding labels.

## 4. Experimental Setup and Implementation

**Figure 4:**
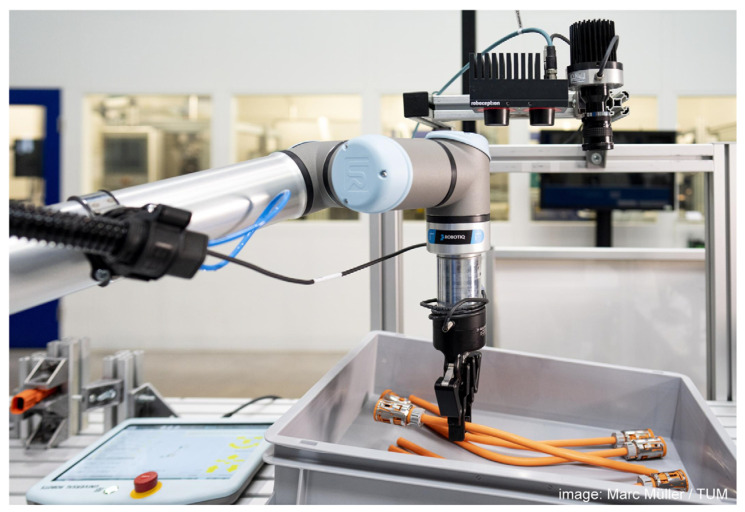
Illustration of the industrial use case of providing DLOs in a small load carrier to a robotic system.

We consider two use cases for the experimental setup of our approach: First, the industrial use case of provisioning DLOs in small load carriers that we have introduced in [30] (Figure 4). Second, a publicly available dataset introduced by Zanella et al. [12], that consists of automatically generated training data and manually annotated real-world images of electric cables. In Section 4.1 and Section 4.2, the experimental setups for both use cases are explained in detail on the basis of the four-step procedure introduced in Section 3. Therefore, we describe the synthetic data generation, test data collection, model training, as well as model test and evaluation metrics for both experiments in the following.

### 4.1. Provision of DLOs in Small Load Carriers

This experiment aims to validate the automatic data-generation pipeline for DLOs and to compare the four replication types for DLOs introduced in Section 3.2. For this comparison, datasets are generated with all replication types. The real-world test data and the subsequent steps are constant kept to compare the results of instance segmentation.

In this experiment, we consider the use case of the provision of DLOs in a small load carrier for an automatic handling or assembly system that requires instance segmentation for consequent grasping [30]. The setup consists of a camera statically mounted with a perpendicular view onto the workbench. Multiple load carriers are provided one after the other on the workbench within the camera’s field of view and filled with control cabinet cables, which are arranged in one of the three scenarios. These cables vary in diameter, length, color, cable ends, and deformation.

#### 4.1.1. Training Data Generation

The automatic data-generation pipeline presented in Section 3 is implemented in Blender using the Cycles engine and executed on a local workstation. We generate one dataset for each of the three scenarios by means of each representation type. These twelve datasets consist of 8000 images each, of which 6400 are used for training and 800 for validation. In addition, 800 synthetic test images, which are not applied in the following, are available in the dataset.

For the visual replication, new DLO models with individual shapes could be generated for each image. However, for practicality, we generate 200 individual geometric models once and randomly select from these for each scene. Analogous to the visual replication, we generate 200 initial shapes for the physical replication, which undergo a scene-dependent deformation in the simulation. For the minimal and reduced replication, only one geometric model is generated and applied in the scenes. The required parameters for each replication type (Table 1) are determined by user input to make the synthetic datasets fit the real-world scenarios.

On this basis, 2000 semi-randomized scenes are simulated for each scenario and each replication type. During the simulation of a scene, keyframes can be stored at various points in time. Thus, several keyframes with a different but similar geometric arrangement can be derived per simulation run. We extract two keyframes from one simulation. In addition, a 180-degree rotated copy of each keyframe is generated. From this in a total of 8000 keyframes we render 8000 images. Rendering of images from the same scene takes place independently, so that different colors and textures can be assigned to the objects. Table 2 describes the synthetic datasets’ properties for all three visual replication scenarios.

For the physical replication of S2 the free fall of DLOs is not implemented in the scene generation due to potential overlapping of the soft body models. Therefore, the models for S2 are spawned directly in the box.

**Figure 5:**
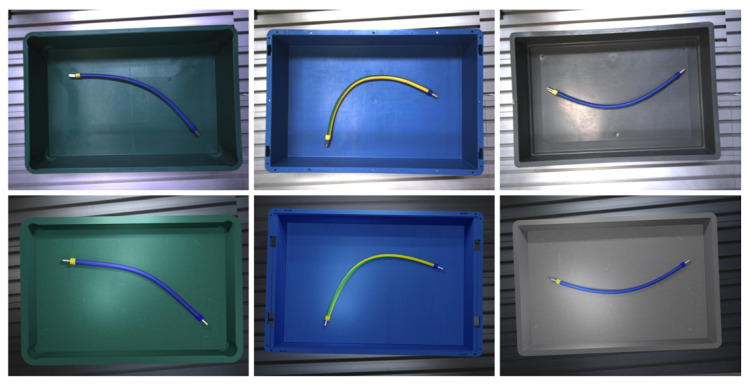
Exemplary comparison of real-world test images (**top row**) and synthetic training images (**bottom row**) generated by the visual replication for the scenario S1.

Based on the findings of [1], we apply textures and colors to mimic the real-world load carriers and DLOs. In addition, real-world images from the workbench are applied as background during rendering. Further, rendering parameters are randomized within a defined range that is kept constant for all datasets. Figure 5 compares real-world test images and synthetic training images from the first scenarios.

#### 4.1.2. Test Data Collection

Labeled real-world images of representative scenes are required to test the trained models for realistic applications. To generate test data, physical scenes of load carriers and DLOs are set up, and photos of the scenes are recorded and labeled manually. We generate one labeled test dataset with control cabinet cables for each scenario.

The photos are recorded using a Roboception rc-visard 65 sensor with an image size of 1280 × 960 pixels. An automatic mode is selected for the white balance and exposure settings. The instance masks are drawn manually into the images for each DLO using a polygon tool (https://github.com/wkentaro/labelme (accessed on 14 November 2022)). Each cable, including cable ends or connectors, is understood as one holistic object marked by one common mask label. About 80 images are available for each of the three scenarios for the use case of the control cabinet cables. We provide the labeled image data in [31].

#### 4.1.3. Model Training

We train a SOLOv2 model [32] with a Resnet101 backbone pre-trained on the COCO dataset [5]. Therefore, we rely on the implementation in the mmDetection framework (https://github.com/open-mmlab/mmdetection (accessed on 14 November 2022)) and keep their configuration to a large extent. We train the SOLOv2 model individually for each of the 12 synthetic training datasets. Thereby the model is trained for ten epochs for the datasets of S1, 15 epochs for the datasets of S2, and 20 epochs for S3. The following augmentation methods are applied during training and testing: resize, random flip, normalization, and padding.

#### 4.1.4. Model Test and Evaluation Metrics

To evaluate the performance of the trained models, we test them on the unseen real-world test data. For this purpose, the test dataset describing the same scenario as the training data is used in each case. Following the predictions of the trained models on the test data, they are compared against the ground truth labels. We compute the metrics for instance segmentation from the COCO dataset [5] evaluation: Average precision (AP) and average recall (AR). Therefore, the segmentation results are classified upon the intersection over union (IoU) between the predicted and the actual segmentation mask. The average precision is computed at an IoU threshold at 50% for AP_0.5_. The AP_0.5:0.95_ and AR_0.5:0.95_ are averaged based on the APs and ARs for IoU in the range from 50% to 95% with a step size of 5%. For the AR_0.5:0.95_ a maximum of 100 detections is considered. The results, which follow from this experimental description, are presented in Section 5.1.

### 4.2. Electric Cable Benchmark

This second experiment aims to validate our automatic data-generation pipeline in another use case. In addition, this experiment aims to compare our approach with the existing cut–paste method for electric cables [12]. For this purpose, one training dataset generated by each method is used to train the same model once each and subsequently evaluate both models on the same test dataset. Thus, the results of both methods can be compared under the same boundary conditions on the same test dataset. The dataset of Zanella et al. [12] contains only semantic mask annotations due to the limitations of the underlying method. Therefore, a semantic segmentation model is chosen for this experiment. The considered use case shows electric cables of different types in highly variable scenes, which also show highly-featured backgrounds and distractor objects, industrial-like settings, and household scenes.

#### 4.2.1. Training Data Generation

The generation of the synthetic training data using the cut–paste method is described in Section 2.2.1 and in detail in [12]. The resulting dataset (https://www.kaggle.com/datasets/zanellar/electric-wires-image-segmentation (accessed on 14 November 2022)) is publicly available for download. The training dataset contains synthetically generated images and about 10% real-world source images. The backgrounds in the synthetic images show low- and high-texture images partially with geometric shapes. Some of the backgrounds appear similar to the backgrounds in the test dataset. Distractor objects are not used in this training dataset. We randomly select 5000 images from the dataset for the training.

**Figure 6:**
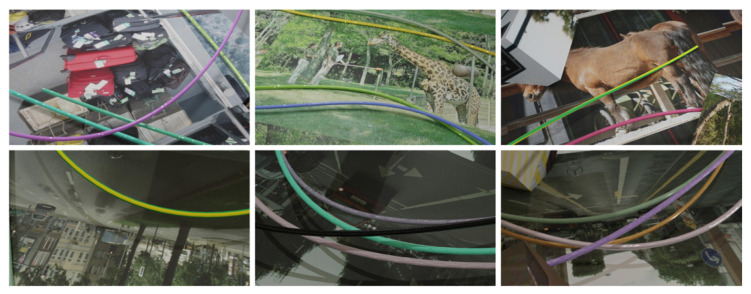
Exemplary images generated with our method for the electric cable benchmark.

In order to generate the second dataset, we apply our proposed automatic data-generation pipeline. Based on the preceding examination, only the visual replication is applied in this experiment. The basic process of data generation is similar to that described in Section 4.1.1. Nevertheless, some parameters are adapted from the first experiment to meet the boundary conditions of this use case. Therefore, no models of small load carriers are applied, and cables are randomly placed in varying numbers within the scene to generate image data of highly variable scenes. Besides, multiple types and sizes of cable ends and connectors are applied to the DLOs. To apply distractor objects and rendering, we build again on the findings of [1]. Therefore, we generate varying-sized cuboids and place them randomly within the scene to partially occlude the cables. From each simulation, three keyframes are exported, and nine images are rendered from different viewpoints. During rendering, images from the Pascal VOC dataset [7] are applied to the cuboids to generate a variation in the distractor objects. Because no background images from the test domain are available, we use images from the COCO [5] and the Cityscapes dataset [33] as background images. During post-processing, Gaussian blur with varying kernel size and motion blur are applied to the rendered images (Figure 6). Finally, semantic segmentation mask labels are computed from the scenes. In line with the test data, our labels map only the deformable part of the cables, so cable ends and connectors are not considered in the binary segmentation mask. Table 3 shows an overview of the major properties of both datasets used for training and the underlying methods.

#### 4.2.2. Test Data Collection

In addition to the synthetic training data, Zanella et al. [12] provide manually annotated real-world test data of different use cases with semantic labels. We use all 62 test images of the dataset, which includes simple scenes without distractor objects, cables in front of highly textured backgrounds, and scenes containing cables, industrial distractors, and household objects.

#### 4.2.3. Model Training

We choose the proven model DeepLabV3+ for semantic segmentation. Thereby, we build upon an implementation (https://github.com/nikhilroxtomar/Human-Image-Segmentation-with-DeepLabV3Plus-in-TensorFlow (accessed on 14 November 2022)) that provides the model architecture with a ResNet50 backbone that is pre-trained on the ImageNet dataset [6]. We train the model for both training datasets individually for 10 epochs. The batch size is set to 2 and a learning rate of $10^{-6}$. In addition, augmentation, including horizontal flip, image rotation, channel shuffle, and coarse dropout, is applied. The model outputs a binary segmentation mask differentiating pixels of DLOs and pixels of other classes.

#### 4.2.4. Model Test and Evaluation Metrics

Finally, the trained models and the real-world test images are inferred. The semantic segmentation performance is evaluated by comparing the output mask with the ground truth mask. Therefore, the IoU between the prediction and the ground truth label is computed. The IoU is used directly as an evaluation metric called the Jaccard index for semantic segmentation. The results that follow from the experimental procedure based on this description are reported in Section 5.2.

## 5. Results

In the following, quantitative and qualitative results obtained from the experiment based on the experimental setup (Section 4) are shown. Section 5.1 summarizes the results for the first use case, where DLOs are provided in small load carriers. In Section 5.2 the results of the electric cable benchmark are shown.

### 5.1. Provision of DLOs in Small Load Carriers

This section presents the quantitative and qualitative results of the experiments described in Section 4.1. These results arise from the inferences of the models, which were trained exclusively on synthetic data with real-world test data. Table 4 shows the quantitative results of the four replication types for the three scenarios, with the best result of each scenario and metric highlighted in bold.

For the scenario S1, all four replication types achieve similar results in each evaluation metric. Throughout, AP_0.5_ is greater than 95% and AR_0.5:0.95_ better than 60% for all replication types. In the second scenario, the results largely deteriorate. The minimal and the physical replication are most affected by this, while the visual replications shows the best results with an AP_0.5_ of more than 90% and an AR_0.5:0.95_ about 60%.

For scenario S3, the metric values deteriorate further and the spread between the results of the replication types increases. The visual replication shows the best results with 70% for the AP_0.5_ and about 45% for the AR_0.5:0.95_. Compared to this, the results of the other replication types are significantly lower. Overall the relative metric gap from S2 to S3 is larger than the one from S1 to S2.

**Figure A1:**
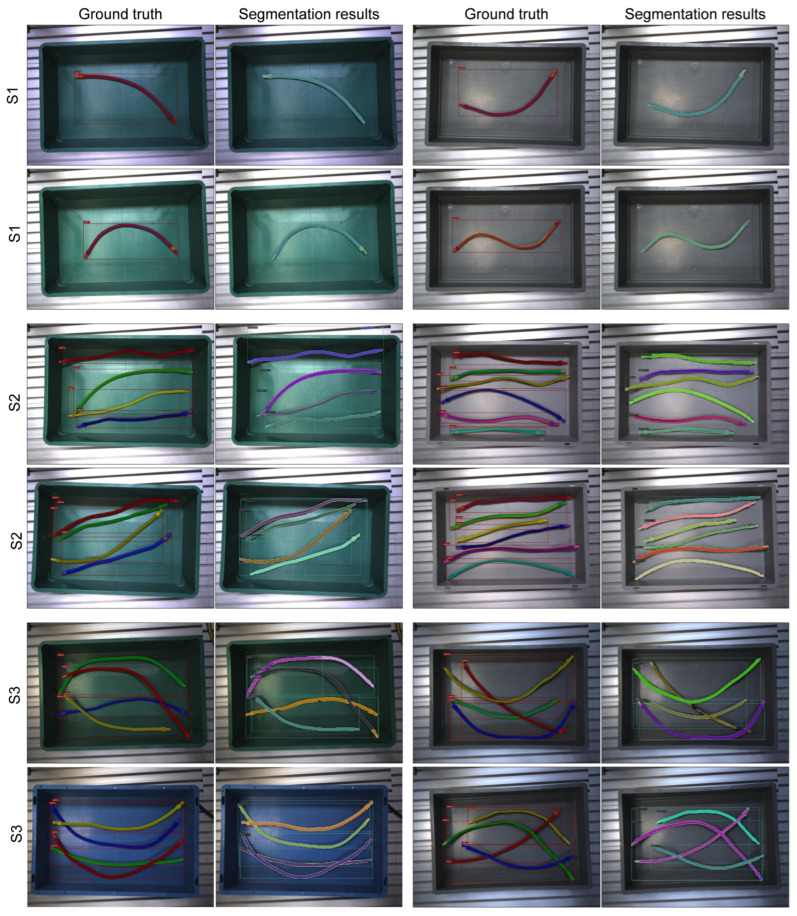
Ground truth masks and instance segmentation results for the visual replication in the scenarios S1, S2 and S3.

Figure A1 shows exemplary results of the instance segmentation for the three scenarios based on the visual replication. For scenario S1, all cables are robustly detected by means of visual replication. Area-wise small errors rarely occur in the shadows of the cables or when a cable is in the shadow of the box. For S2, the pixel-wise classification between cable and background is predominantly correct. Only few pixels, e.g., the slots of the background table, are classified as false positives. For the majority, a complete mask is output for the cables. In a few cases, the mask segments are incorrectly assigned for cables that lie close together. For S3, cable edges are segmented correctly in many places and correct masks are output for cables lying on top. Some other cables are divided into several small mask segments, which in sum describe the correct cable shape.

**Figure A2:**
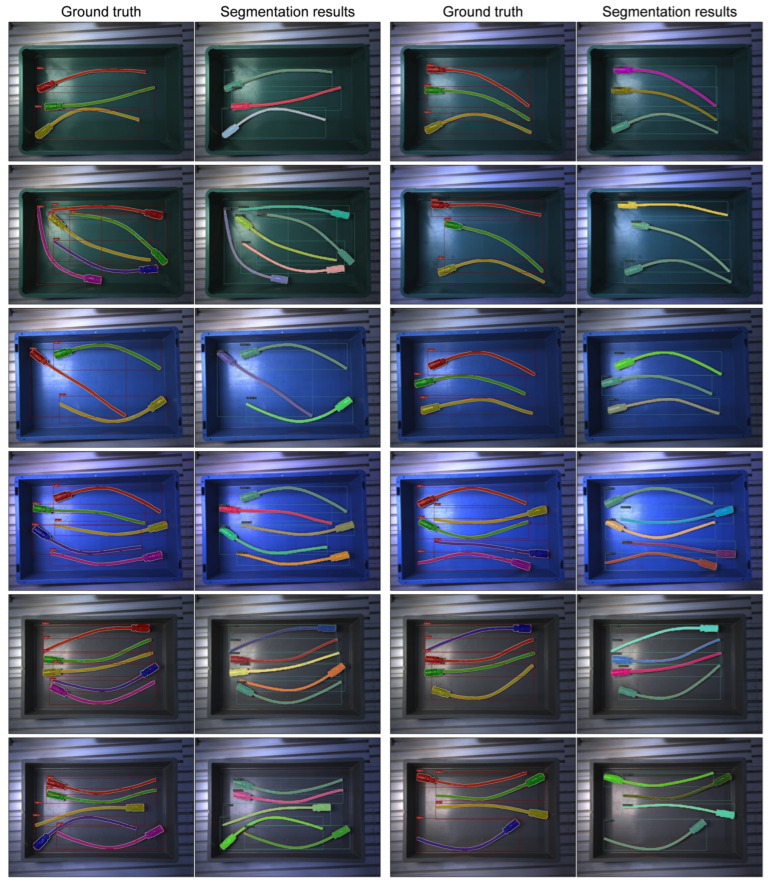
Ground truth masks and instance segmentation results for high-voltage cables in the scenario S2.

**Figure A3:**
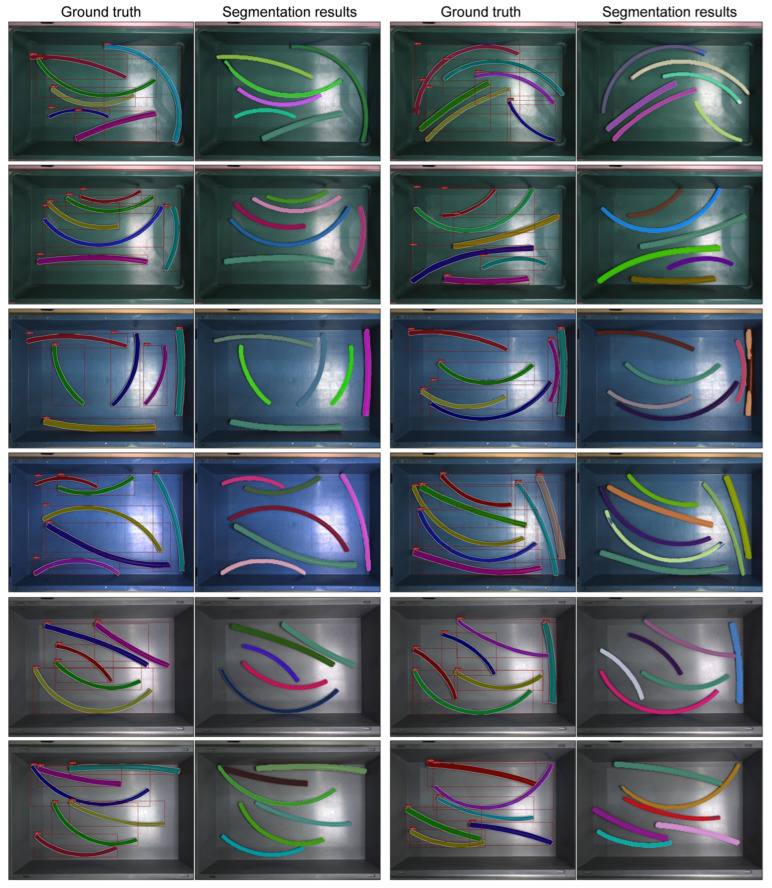
Ground truth masks and instance segmentation results for pneumatic hoses in the scenario S2.

In addition to the previously described experiments with control cabinet cables, we apply the same experimental setup to other DLOs to examine the transferability of the automatic data-generation pipeline. Therefore, we choose high-voltage cables from the automotive industry and pneumatic hoses in the scenario S2. The high-voltage cables have larger connectors on one end, while the pneumatic hoses have no connector attached. For these DLOs, the steps training data generation, test data collection, model training, and model test are executed in the same manner as for the control cabinet cables. The main difference is that only the visual replication is used due to the previous results. Additional real-world test images are used for a qualitative evaluation. The ground truth masks and resulting images for the high-voltage cables (Figure A2) and pneumatic hoses (Figure A3) are shown in the Appendix A.

For the high-voltage cables, which are identical in their geometric dimensions but different in their deformation, the pixel-wise classification is predominantly correct. In some small areas, a deviation of the step-like mask from the mostly round cable shape can be seen. For the pneumatic hoses, despite the diversity in terms of shape, color and texture, correct masks are output except for a few small areas.

### 5.2. Electric Cable Benchmark

**Figure A4:**
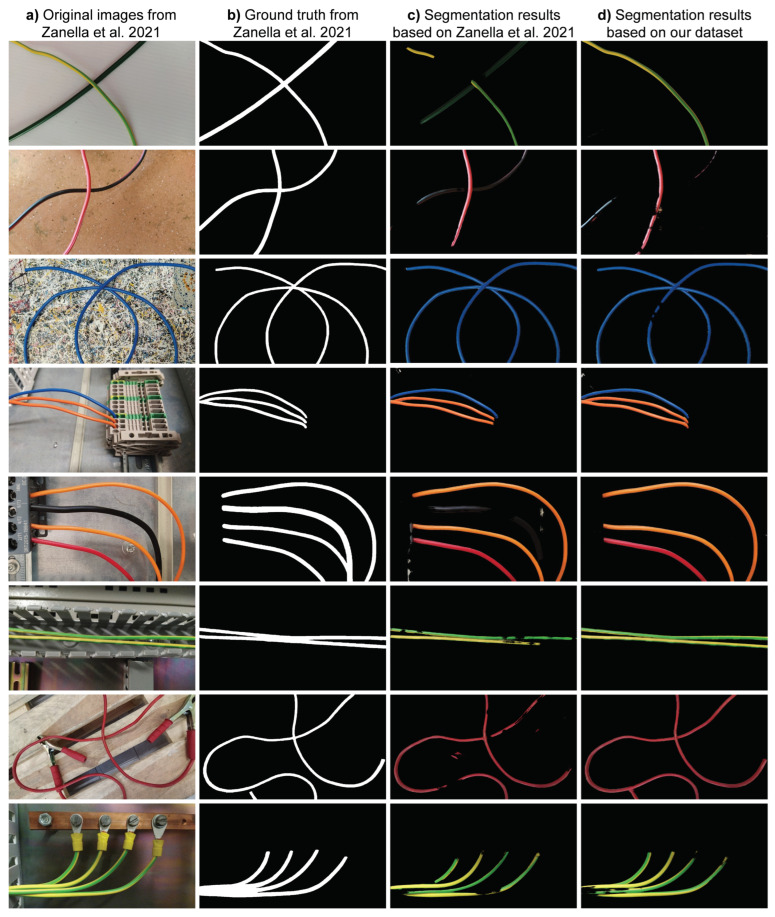
Semantic segmentation results for the electric cable benchmark: (**a**) Original images from [12], (**b**) ground truth segmentation masks from [12], (**c**) results based on the dataset of [12] and (**d**) results based on our dataset.

In the following, the results from the electric cable benchmark are shown. Table 5 presents the quantitative results for the semantic segmentation, once for the dataset based on Zanella et al. [12] and once for the dataset based on our data generation approach. The evaluation metrics show comparable results with minor differences for both datasets. A qualitative evaluation of all test results shows that the dataset based on [12] provides better results for dark and dirty cables than ours. Moreover, the segmentation results for test images with background patterns highly similar to the training images from [12] are better than our results. Nevertheless, with our training dataset, it is possible to detect cables with shapes, arrangements, and colors, which in some cases differ strongly from the training data. In addition, the segmentation results of our dataset are better for two-color cables, for example. Figure A4 shows exemplary semantic segmentation results for both datasets in comparison with the original images and the ground truth annotations.

## 6. Discussion

In the preceding experiments, the data pipeline generates training data for multiple DLOs and scenarios. The segmentation results show the feasibility of the presented pipeline across multiple use cases. The data-generation pipeline requires only an initial user input for parameterization. Therefore, this simulation and rendering method does not require geometric models as input. Instead, DLO models are generated in several ways. The manual work mainly concerns the selection of object textures and DLO-related parameters. Consequently, the effort depends on the selected replication type but is independent of the number of images to be created.

The visual replication is the most effective of the examined replication types. It shows overall the best results for instance segmentation, while fewer user input parameters are required compared to the physical replication. The reason for the better segmentation results seems to be the variance of the geometric shapes of the DLOs, which does not exist for the minimal and reduced replication. The physical replication is hardly visible for the scenarios S1 and S2 because there are no overlaps between DLOs. In addition, the DLOs for S2 are spawned directly in the box, which leads to less randomization in the arrangement. In scenario S3, the physical replication cannot achieve the best results despite the higher degree of realism. A possible reason for this may be that the physical deformation effects appear relatively small in the images. In summary, the physical replication results in medium segmentation performance, despite the most input parameters.

From the comparison of replication types we conclude that, on the one hand, no soft body simulation of DLOs is required for the generation of synthetic image data. On the other hand, mapping the geometric variance of the deformation is a crucial factor in improving the segmentation results. Thus, we recommend modeling DLOs as rigid body models with visual replication to generate synthetic training data.

If unseen synthetic images are used for the model test, this results in considerably better results than real-world images. For example, an AP_0.5:0.95_ greater than 80% is achieved for S2 with the visual replication. Therefore, we conclude a significant domain gap between synthetic and real-world images. The domain gap with respect to rendering is not a DLO-specific problem and thus not scope of this paper.

The introduction of the three different scenarios for the arrangement of DLOs allows us to classify future use cases and test scenarios and thus establish better comparability. This is advantageous because the segmentation results for the three scenarios show how different the performance can be for various arrangements. While for S1 and S2 most DLOs are segmented with correct masks, for S3 DLOs are often described by several submasks. The quality of the S3 masks requires further post-processing steps to make it usable for robotic grasping.

The automatic data-generation pipeline is applicable to various use cases and flexible for the adaptation to new DLOs. Thus, various DLO configurations and scenarios can be generated based on the initial user input. In addition, new DLO types can be generated by adding new models of cable ends and connectors or adjusting the geometric and deformations parameters to modify the DLO body. The transferability is shown by examples of high-voltage cables and pneumatic hoses. For both DLO types qualitative results comparable to the control cabinet cables or better are achieved. In addition, the pipeline can generate generic datasets without a specific industrial use case.

The electric cable benchmark shows that datasets with high variability can also be generated by employing our automatic data-generation pipeline. Further, it is demonstrated that the simulation and rendering method can achieve comparable results to the cut–paste method. This is remarkable, as the dataset from [12] contains source images of DLOs and backgrounds similar to the target domain, as well as about 10% of real-world training images. In contrast, our approach purely trains on synthetic DLO images. As described in Section 5.2, the segmentation results based on the two training datasets are dependent on the boundary conditions such as cable color and background. The better performance based on the data from [12] for highly cluttered backgrounds can be explained by the fact that similar background images are used in the training and test dataset. On the other hand, the explicit representation of two-color cables in our training dataset results in superior performance for this color pattern. However, these differences cannot be fundamentally attributed to the basic functions of the two methods. This is because both methods receive different inputs, for example with regard to the distribution of the cable color, and thus result in differently balanced datasets. A comparison of results for individual features is not with in the scope of this section.

Considering the comparable performance of both approaches, our automatic data generation offers advantages regarding reduced manual effort for the scene generation and the flexibility regarding model and scene generation. Nevertheless, it should not be disregarded that our method requires manual effort for the user input and the selection of suitable textures. A generalized comparison of the segmentation performance and manual efforts of the two methods for generating synthetic training images is not possible at this point.

Finally, we address the methodological limits of our work structured by the steps of our procedure. Firstly, we do not specifically investigate the domain gap regarding object textures and rendering but build up on the findings of [1]. Fine-tuning object textures and rendering properties will likely affect better segmentation results on real-world test data. Secondly, the manual image annotation of ground truth labels is always subject to imprecision. In addition, the confined amount of real-world test images limits the statistical significance of our results. Thirdly, we use off-the-shelf deep learning models for general object segmentation and do not optimize model parameters and training. Fine tuning is likely to produce better results.

## 7. Conclusions

The full potential of deep-learning-based localization can be exploited only if sufficient high-quality training data is available. Synthetic image data is a cost-efficient possibility to create specific datasets for industrial applications.

In this context, we address the lack of training data for deformable linear objects. We present an automatic image generation pipeline for DLOs to enable instance segmentation of various cables and pneumatic hoses. The user input of the pipeline aims at a simple and flexible transfer to other DLOs and use cases. The data-generation pipeline is validated for multiple DLO types in the use case of bin picking. Thereby, we show that visual replication is best suited for modeling DLOs. For unoccluded DLOs mostly complete segmentation masks are achieved. A larger domain gap occurs for crossed cables, which shows up in partially subdivided or incomplete masks. The introduction of reference scenarios for scenario complexity allows a comparison of the results for different DLO arrangements. A further comparison shows that our method can produce comparable results to the cut–paste method with little manual effort.

In future work, we will further investigate synthetic data generation and instance segmentation of DLOs especially for the scenario S3. In addition, we will integrate the localization of DLOs into a robotic system to evaluate the segmentation performance using robotic grasping operations.

## Figures and Tables

**Table 1:** Overview of the application of geometric, deformation and soft body parameters for the replication types with discrete values (x) and ranges of values (o).

Replication Type	Geometric Parameters	Deformation Parameters	Soft Body Parameters
Minimal replication	x	-	-
Reduced replication	x	x	-
Visual replication	o	o	-
Physical replication	o	o	x

**Table 2:** Properties of the synthetic datasets generated by the visual replication for the scenarios S1, S2 and S3.

Dataset Properties	S1	S2	S3
Image size [pixels]	1280 × 720	1280 × 720	1280 × 720
Number of images	8000	8000	8000
Number of cables per image	1	[4, 6]	[4, 6]
Number of crossings per image	0	0	≥0

**Table 3:** Properties of the synthetic training datasets based on [12] and our method.

Dataset Properties	Dataset Based on [12]	Dataset Based on Our Approach
Data generation method	cut–paste	simulation and rendering
Scene generation	manual	automatic
Number of images	5000	5000
Image size [pixels]	min. 1280 × 720	1280 × 720
Distractor objects	none	cuboids
Cable texture	target domain	synthetic
Background images	manually selected	open source dataset

**Table 4:** Results of the inferences for all replication types and scenarios for control cabinet cables. The best result of each scenario and metric is shown in bold.

Scenario S1	AP_0.5_	AP_0.5:0.95_	AR_0.5:0.95_
Minimal replication	0.960	0.588	0.662
Reduced replication	**0.979**	0.604	0.660
Visual replication	0.970	0.646	0.694
Physical replication	0.966	**0.661**	**0.713**
**Scenario S2**	**AP_0.5_**	**AP_0.5:0.95_**	**AR_0.5:0.95_**
Minimal replication	0.797	0.312	0.423
Reduced replication	0.810	0.372	0.503
Visual replication	**0.979**	**0.574**	**0.637**
Physical replication	0.663	0.262	0.399
**Scenario S3**	**AP_0.5_**	**AP_0.5:0.95_**	**AR_0.5:0.95_**
Minimal replication	0.345	0.075	0.199
Reduced replication	0.618	0.233	0.365
Visual replication	**0.706**	**0.332**	**0.453**
Physical replication	0.406	0.162	0.259

**Table 5:** Semantic segmentation results for datasets based on [12] and our approach.

Evaluation Metric	Dataset Based on [12]	Dataset Based on Our Approach
Jaccard index	0.702	0.675

## Data Availability

The data presented in this study is partially available in mediaTUM at [31]. Further data of this study is available on request from the corresponding author.
